# Demystifying the mist: Why do individuals hesitate to accept AI educational services?

**DOI:** 10.1111/bjop.70040

**Published:** 2025-11-10

**Authors:** Aiping Shao, Zhi Lu, Stephanie Q. Liu, Yin Shi, Wei Lu

**Affiliations:** ^1^ School of Educational Science Beihua University Jilin City Jilin Province China; ^2^ Antai College of Economics and Management Shanghai Jiao Tong University Shanghai China; ^3^ School of Education & Human Development Texas A&M University College Station Texas USA; ^4^ College of Education and Human Ecology The Ohio State University Columbus Ohio USA; ^5^ Faculty of Education Beijing Normal University Beijing China

**Keywords:** AI educational services, attribution, conformity, guilt, valuation, word‐of‐mouth

## Abstract

Rapid advances in AI technology are fuelling the proliferation of AI applications across industries, including educational services. With the allure of intelligent tutoring, individuals now face the choice of their educational approach—either parental engagement or utilizing AI educational services. This research employs an experimental design approach to examine individuals' decision‐making processes involving AI educational services. Across five studies, we observe that, relative to AI educational services, parental engagement induces less guilt, receives a higher valuation and increases individuals' willingness to recommend it to others. We attribute these preferences to a perceived parental responsibility. Intrinsic attribution and conformity promote individuals' WOM. This research is the first to uncover the impact of educational approaches on individuals' guilt and downstream behaviours in the AI‐in‐Education field, shedding light on attribution as its underlying mechanism and offering actionable strategies to enhance individuals' WOM. The findings offer novel insights to AI‐human interaction psychological research and hold practical implications for AI‐in‐Education industry practitioners.

*“That best academy, a mother's knee*.”

*— James Russell Lowell*

## BACKGROUND

The rapid progress in artificial intelligence (AI) has profoundly impacted nearly every aspect of today's world, including the education landscape. Due to its interactive learning approach and its ability to transcend time and place constraints, the use of AI is becoming increasingly popular (Zhou et al., 2020). According to Global Market Insights (2023), the revenue generated by the AI educational service market is predicted to exceed $30 billion by 2032, underscoring the vast potential of the burgeoning AI‐in‐Education industry. As such, pioneering AI education companies like Khan Academy, Cognii and KidSense market their AI educational services by positioning them as intelligent tutors capable of assuming the role of teaching or coaching students, thereby enabling accurate and effective learning. One company, in particular, promotes its AI educational services with the slogan, “Let the children learn how to learn, parents' inputs are not required.”

Nonetheless, parental engagement plays a vital role in a child's education, which has been proven to be an effective way to improve students' academic achievement. Recent research from the *National Coalition for Parent Involvement in Education* solidly supports that the extent of parent involvement is the most accurate predictor of student success in school, including their test grades, social skills, confidence and adaptive behaviour (National PTA, 2019).

Therefore, the key questions raised by this current research are as follows. In the context of AI educational services and parental engagement, which option do individuals prefer and why? Our rationale for this focus is rooted in several key factors. Firstly, the majority of parents recognize that a child's future largely hinges on the education he or she receives. Therefore, parents are motivated to go to great lengths to ensure the best possible education for their children. This may involve embracing cutting‐edge educational technologies such as AI educational services or dedicating substantial time and efforts to personally guide their kids in learning and development. Secondly, AI‐powered services in K‐12 education constitute the core market for current educational tech companies, contributing a substantial portion to their profits. These services offer students key advantages, such as homework support, personalized learning and skill development (Labadze et al., 2023). However, research on the application of AI in education remains nascent. To date, most studies have relied on pre‐/post‐tests designs or self‐report questionnaires to gauge effectiveness, with scholars predominantly comparing different chatbot systems in pursuit of optimal implementation strategies. Nevertheless, empirical work situated in K‐12 settings is particularly scarce and rigorous investigations into educational phenomena such as learning behaviours remain limited (Hwang & Chang, 2023). In practice, users are concerned about issues such as information sources, students' initiative in learning and the uncertainty of biases and misinformation (Han et al., 2024). Individuals' attributions regarding AI efficacy are shaped by their understanding of AI's pedagogical advantages and limitations (Chen et al., 2025). Consequently, widespread adoption and effective integration of AI in education require further scholarly and practical advancement. Of note, parents play a pivotal role in determining the educational approach adopted for their children during this stage. Thirdly, the education parents provide for their children carries symbolic significance. It serves as an embodiment of parents' love for their children and parents' responsibility of educating their children, which often times is shared and passed down through generations via social norms and values.

At present, the “emotional‐cognitive framework”—widely adopted in psychology and AI—integrates the emotional and cognitive dimensions to provide a more comprehensive understanding of human behaviour and the complex phenomena in human–computer interaction. The emotional dimension encompasses emotional experience, motivations and subjective feelings (e.g., technology anxiety, Kim et al., 2023; stress, Rasouli et al., 2024), whereas the cognitive dimension involves information processing, reasoning, memory and knowledge structures (e.g., trust in AI, Klingbeil et al., 2024; Leschanowsky et al., 2024; Rheu et al., 2021; perceived usefulness, Ling et al., 2021; Pitardi & Marriott, 2021; and technology self‐efficacy, Kulviwat et al., 2014). For instance, when an AI system demonstrates cognitive and behavioural competence, users are more likely to place trust in it (Salem et al., 2015). Although the “emotional‐cognitive framework” has been employed to explain why individuals adopt AI, few studies have examined the downstream emotions or behaviours resulting from people's use of AI.

As the major decision makers of AI educational service, parents have two different approaches to consider when making choices for their kids' education. Some parents are inclined to embrace the AI educational service given it not only assists their children in achieving better outcomes in their learning but also alleviates parents' stress and workload associated with children's education. In contrast, other parents hold a contrasting perspective. They assert that the responsibility of educating children lies squarely on the shoulders of parents and should not be delegated to machines. This group of parents contends that any parents who opt for the AI educational service may ultimately grapple with feelings of remorse when facing their children.

Despite the fact that most educational service decisions are made by parents for their kids and that parents are the actual buyers of AI educational services, surprisingly, few studies on nascent AI educational services have investigated parents' psychology and behaviours, particularly their emotions (e.g., guilt) and downstream behaviours such as valuation and word‐of‐mouth (hereafter WOM). Both factors are crucial to the success of AI educational services: the former reflects individuals' perception of the value of AI educational services, while the latter captures their willingness to actively share the benefits of AI educational services with others.

In the current research, we are intrigued to delve into the impact of individuals' educational approach (using AI educational services vs. parental engagement) on their feelings of guilt towards their children and how such feelings may influence their downstream behaviours, including valuation and WOM. Furthermore, we aim to unravel why individuals respond differently to these educational approaches and disclose effective strategies for AI educational service companies to reduce individuals' guilt and improve their WOM.

To sum up, this research fills the lacuna in individual responses for AI educational service and explores individuals' feelings of guilt and consequent behaviours when using AI educational services. In addition, few studies focus on the intrinsic attribution and conformity in the context of AI educational services, and how these factors influence individuals' WOM. In this research, we explore these aforementioned questions and address an important research gap in AI and human interactions through the lens of individual psychology. To illustrate, this research advances previous studies in the following three key domains.

First, this research is the first to reveal the emotional dark side of using AI educational services, broadening our understanding of the negative emotions activated by using AI educational services (Brusoni & Vaccaro, 2017; Martin et al., 2019). Despite the functional advancements in AI, there is a noticeable oversight regarding the potential for individuals to experience adverse emotional states when interfacing with AI educational services. These negative emotions could cause individuals to hesitate in adopting and utilizing AI educational services. Considering that the ultimate success of AI relies on individual acceptance and utilization (Becker & Jaakkola, 2020; Moore, 2014), and emotion may play a crucial role in influencing individuals' adoption decisions. Our research on individuals' emotional responses to AI educational services highlights a key point: industry practitioners should understand the potential emotional impact of AI on parental individuals and develop strategies to mitigate negative emotions and behaviours associated with AI services.

Second, our research explores individual preferences between AI educational services and parental engagement, shedding light on the motivations behind these choices. This is essential for deciphering the psychological and behavioural aspects of parental individuals in the burgeoning AI educational services market. As guardians and the major decision makers of their children's AI educational services, parents need to balance between the benefits that AI educational services provide and the nuanced social norm of their parental responsibility as conscientious caregivers. Garcia‐Rada et al. (2022) indicate that individuals are more willing to choose the more effortful way to take care of others, thus symbolically demonstrating their love for others. Our line of theorizing and accompanied empirical evidence demonstrate that this social norm‐driven motivation induces guilt in parents, which further discourages them from supporting AI educational services or advocating their benefits. This finding highlights an intriguing yet hitherto under‐examined attribution psychological mechanism that influences individuals' responses to AI. It provides valuable insights for AI service providers to develop effective strategies addressing the intrinsic needs tied to parental responsibility. This, in turn, mitigates the negative ramifications of individual disengagement and strengthens AI's value in education and other relevant industries.

Third, our research examines an array of diverse and coherent parent responses towards AI educational service, spanning feelings of guilt, valuation and WOM. This comprehensive investigation corroborates one another so that we expand beyond the normal focus in psychology research and achieve a panoramic understanding of individuals' responses. Of note, WOM is a significant post‐consumption behaviour as it not only helps individuals make better consumption decisions (Hennig‐Thurau et al., 2004) but also affirms a positive image of one's ability to influence others (Barasch & Berger, 2014). Given the online nature of AI educational services, WOM is crucial for their dissemination and reputation among interested individuals. Furthermore, our research explores the role of intrinsic attribution and conformity in shaping individuals' WOM behaviour. This provides actionable insights for AI service providers to enhance individuals' WOM, which can consequently improve the overall service experience of using AI services.

## THEORETICAL BACKGROUND

### AI educational services

Driven by the rapid advancements in AI technology, AI educational services offer a range of impressive advantages over human tutoring. Firstly, AI educational services significantly reduce the cost of education, including the tutoring efforts of teachers and parents. Secondly, AI educational services provide students with comprehensive feedback, step‐by‐step solutions and suggestions for improvement while also proposing ideas for further learning. This enables teaching strategies to be tailored to each student's unique needs (e.g., Celik et al., 2022; Crawford et al., 2023; Fariani et al., 2023; Fauzi et al., 2023; Kikalishvili, 2023; Lo, 2023; Qadir, 2023; Schiff, 2021; Shidiq, 2023). Thirdly, AI educational services contribute to mitigating the imbalance of educational resources and levelling the playing field for underprivileged children who lack access to quality education. Due to these advantages, AI educational services have become the next blue ocean market for AI technology and one of the hot trends in the recent AI landscape.

At present, many studies have explored how different forms of AI can enhance children's learning in various ways. For example, in hybrid or collaborative parent–AI educational practices, AI functions not merely as a learning tool but also as a learning partner, participating in the educational process alongside parents, who play a pivotal intermediary role. Specifically, parents act not only as consumers of AI‐generated outputs but also as bridges connecting AI systems with their children. Conversational agents, for instance, can serve as joint reading partners, engaging children in scaffolded, story‐related conversations (Xu & Warschauer, 2020). AI systems can also generate adaptable teaching materials, foster creativity and provide students with personalized and timely feedback (Han et al., 2024). Moreover, AI helps parents better understand their children's learning needs and provide tailored support. For example, the design of StoryBuddy emphasizes dynamically accommodating parental involvement while safeguarding parent–child relationships and minimizing the need for parental intervention during busy periods (Zhang, Xu, et al., 2022).

China is one of the pioneers in the development of AI educational services and also one of the largest AI markets in the world. In this research, we focus on Chinese consumers as our research participants due to the following considerations. First, China's market demand is vast, and its market size continues to expand. According to a recent report, the size of China's smart education market is expected to exceed 900 billion yuan by 2025, with a compound annual growth rate of approximately 21% (ChinaIRN, 2024). Second, the number of current consumers of AI educational services is immense, given the strong presence of such services in this market. This provides a rich research context for examining parent‐consumers' responses towards AI educational services. Third, Chinese parents, like parents elsewhere, place great importance on their children's education (Somerville & Robinson, 2016). They also tend to hold higher expectations for academic achievement and invest considerable effort in their children's educational process (Cheung & Pomerantz, 2011), as education is regarded as a key pathway to social mobility (Tam & Chan, 2010). However, they face challenges related to the uneven distribution of high‐quality educational resources, which has further contributed to the growth of AI educational services.

Although AI services provide numerous benefits to individuals and are rapidly improving in functionality, people still exhibit resistance to these services due to various factors, including privacy concerns, incorrect prediction and lack of supervision (e.g., Dietvorst et al., 2018; Longoni et al., 2019). Another potential factor may be the negative emotions triggered by social norms (e.g., Giroux et al., 2022). Parents enact their parental role based on the ideal‐parent belief (Super & Harkness, 2020), which guides their cognition, behaviours and decisions (Hale et al., 2017; Karlsson et al., 2013; Keller, 2012). Lin et al. (2023) suggest that the ideal‐parent belief of Asian parents emphasizes responsibility and a child‐ or family‐centred orientation. When parents perceive their own practices as falling short of this ideal‐parent standard, they may experience guilt (Eaton et al., 2016). Nonetheless, few extant research studies have explored individuals' resistance to AI services, including their emotions and behaviours, especially feelings of guilt.

### Guilt

Guilt is an important negative emotion in individual psychology. It is a negative self‐conscious emotion experienced by individuals when their decisions conflict with personal values or social norms (Agrawal & Duhachek, 2010; Burnett & Lunsford, 1994; Tangney & Dearing, 2002). Many individual behaviours can trigger guilt, such as unethical consumption (Adil, 2022), indulging in delicious food (Mohr et al., 2012) and excessive shopping (Bei et al., 2007). Previous studies have extensively examined the impact of individual guilt induced by marketers on consumption behaviour. For instance, on the one hand, marketers use guilt induction as a marketing strategy to encourage individuals to purchase services (Burnett & Lunsford, 1994; Soscia et al., 2008), or to promote compensatory consumption (Dahl et al., 2005). On the other hand, marketers also work to alleviate individuals' guilt associated with a certain product (e.g., luxury product) through marketing strategies such as associating the purchase with virtuous acts (Khan & Dhar, 2006), emphasizing efforts (Kivetz & Simonson, 2002), or encouraging volunteering (Jeong & Koo, 2015), thereby increasing the likelihood of corresponding service purchase.

Although guilt is a negative emotion, it is generally adaptive (Tracy & Robins, 2006). Researchers believe that guilt is a situational emotion associated with specific individual behaviour (Tangney et al., 2007). For instance, Wolfers et al. (2025) suggest that parents experience guilt when they limit their children's screen use time. Yi and Baumgartner (2011) classify guilt into four categories based on context: financial guilt, health guilt, moral guilt and social responsibility guilt. More specifically, social responsibility guilt arises when individuals' decisions go against personal social norms. For instance, under the social norm that “effort shows love”, individuals are more hesitant to adopt labour‐saving products (Garcia‐Rada et al., 2022). In addition, guilt can be categorized based on its focus – either guilt directed towards oneself or towards others. In a nutshell, our research concentrates on guilt towards others in the context of social responsibility.

### Educational approach and guilt

Education is one of the most important investments in almost every family, and parents try their utmost to help their children in their academic pursuits. Yet, how to help their children excel in the learning journey is still a challenge for many parents. AI educational services could be a significant help for parents to address this challenge given that they offer numerous potentials for both educators and students, including homework support, personalized learning and skill development (Labadze et al., 2023).

Although AI educational services have a number of outstanding advantages in educating students through their well‐designed functions, thereby relieving parents of their tutoring responsibility and saving their time and effort, these AI educational services more or less replace the role of parents in educating their children. From a social norms perspective, parents value their efforts in raising children because these efforts symbolically represent their love for their children and their ability as competent caregivers (Cutright & Samper, 2014; Kruger et al., 2004; Morales, 2005; Olivola & Shafir, 2013). Garcia‐Rada et al. (2022) find that using convenient products can lead individuals to feel that they are not putting in sufficient effort for their children; as a result, individuals are more willing to invest more energy in caregiving tasks. Likewise, Seagram and Daniluk (2002) suggest that the love and affection between parents and children motivate parents to strive for better child‐rearing practices and to set high standards for their parenting.

In line with this reasoning, parents who adopt the AI educational approach (i.e., using AI educational services instead of direct parental engagement) are likely to feel that they are reducing their caregiving responsibilities and not making enough effort to fulfill their roles as parents. Consequently, parents may feel guilty when they believe they are responsible for their children's future but fail to match their own or society's expectations (Rotkirch & Janhunen, 2010). We theorize that when parents use AI educational services to educate their children instead of doing so themselves, they may perceive their behaviour as a potential violation of social norms of parenting and thus feel guilty (Soscia et al., 2008). Thus, we hypothesize:

Individuals are more likely to feel guilty about using AI educational services to educate their children than parental engagement.

### Educational approach, valuation and WOM

According to the UTAUT2 theory, individuals' acceptance and use of new technologies are influenced by social factors (Venkatesh et al., 2012). As human–computer interaction increases, the strong performance of AI increases individuals' trust in AI (Rheu et al., 2021). For instance, Aslan et al. (2024) suggest that after parents experience Kid Space with their children, their concerns about screen time, social interaction and physical activity are significantly reduced, and they develop positive views regarding its educational value.

Prior work attests that individuals' feelings of guilt influence their downstream behaviours. Lee‐Wingate (2009) finds that advertisements highlighting mother–child relationships effectively evoke guilt in working mothers who are unable to spend sufficient time with their children. This heightened sense of guilt often prompts working mothers to engage in compensatory purchase behaviours, attempting to make up for their absences. Alongside compensatory consumption, guilt can also influence individuals' valuation of products and services.

The success of AI educational services cannot be separated from two key factors: one is that individuals' perception of the value of AI educational services (i.e., valuation), and the other is that individuals' willingness to actively share the benefits of AI educational services with others (i.e., WOM). Only when individuals perceive that the value of AI educational services will they be willing to use and pay for them, enabling AI educational service providers to continue iterating and upgrading their AI‐powered technology. This, in turn, allows the entire AI‐in‐Education industry to generate profits, reinvest in new service development and achieve sustainable growth. Similarly, only when individuals are willing to recommend AI educational services to others can more parents become engaged, allowing AI educational services to gain greater popularity among their target individuals.

Valuation refers to individuals' subjective cognition and evaluation of the utility of a given product or service. Given the central role it plays in individuals' decision‐making process, valuation is widely recognized in existing work as a significant factor affecting individual attitude, purchase intention, impulsive consumption, satisfaction, WOM and other behaviours (Chen & Chen, 2020; Han et al., 2021; Liu et al., 2021; Pang, 2021; Yang et al., 2021). For instance, the Ikea effect suggests that individuals tend to overestimate the value of their own efforts when they have invested a significant amount of labour or emotion into a task (Mochon et al., 2012; Norton et al., 2012). This effect is also relevant in the context of education. Seagram and Daniluk (2002) propose that the love and affection between parents and children motivate parents to set high standards for their parenting. Educating children often requires parents to spend substantial resources, such as time and money. As a result, parents may overestimate the value of their own efforts, which can be reflected in their downstream behaviour, including their valuation of AI educational services. Specifically, parents perceive less value in AI educational services compared to their own engagement in their children's education. Thus, we hypothesize:

Individuals value their efforts in parental engagement more than using AI educational services.

Meanwhile, in today's highly connected world, WOM is one of the primary sources individuals rely on for product and service information before making purchasing decisions (Dean & Lang, 2008; Zhang, Sun, et al., 2022). Also, an increasing number of individuals engage in WOM as an effective way to disseminate their post‐purchase information, shopping experiences and knowledge, while also expressing their own social status and uniqueness in online communities (Godes & Mayzlin, 2004; Lee, 2021; Lee & Watkins, 2016; Yusuf et al., 2018; Zhu & Zhang, 2010).

We believe that, compared with parental engagement, individuals are less likely to spread the word to others when using AI educational services. The reasons are as follows. First, in the context of choosing an educational approach, many parents have become accustomed to a long‐established social norm which implies that parents shall educate their children themselves. Those who chose not to do so (e.g., by using AI educational service) may incur feelings of guilt. Soscia (2007) suggests that guilt inhibits WOM. Second, WOM, as a prosocial behaviour (Septianto & Chiew, 2018), is conducive to enhancing the affective connection between recommenders and achieving better interpersonal interaction and exchange (Penner et al., 2005). However, unlike humans, AI does not possess emotional capabilities (Han et al., 2022; Wien & Peluso, 2021), and consumers therefore have no expectations of emotional or resource exchange with AI. Third, consumers believe that AI lacks subjective initiative; even when AI performs well, consumers do not make positive attributions to AI (Garvey et al., 2023; Jörling et al., 2019). Therefore, we theorize that, despite the many benefits of AI educational services, individuals are more willing to educate their children themselves rather than using AI educational services. Similarly, they are more inclined to communicate the benefits of personally educating their children to others compared to using AI educational services. Thus, we hypothesize:

Individuals are more willing to spread words to others about parental engagement than to use AI educational services.

### The mediating role of attribution

Attribution, as proposed by Heider (1958), refers to the reasons that individuals infer for the occurrence of their own or others' behaviours. Later, Weiner (1979) develops attribution theory, in which individuals judge the causes of events along three dimensions: locus of causality, stability and controllability. For example, effort is considered an internal, stable and controllable attribution, while luck is viewed as an external, unstable and uncontrollable attribution. In our research, we focus on how the attribution of different educational approaches influences individuals' guilt and subsequent valuation, as well as WOM.

With the widespread adoption of AI across a variety of industries, individuals increasingly regard AI as a social entity (e.g., Huang & Rust, 2021a, 2021b; Van Doorn et al., 2017; Wilson et al., 2017) and make attributions to AI's behaviours. For instance, when comparing AI to humans, individuals tend to criticize AI service providers less than human providers because AI is perceived to have less control over service outcomes (Belanche et al., 2020; Leo & Huh, 2020).

In recent years, both society and policymakers have encouraged and guided parents to participate more in their children's education (Avvisati et al., 2014). Parental engagement can effectively enhance parent–child communication and children's self‐efficacy (Lau et al., 2012; Mandarakas, 2014). Therefore, although AI is already widely used in education, tutoring children is still largely regarded by social norms as a responsibility that parents themselves should undertake. When parents use AI educational services to educate their children instead of engaging directly, these parents may feel that they have not fulfilled their responsibility to educate their children. This behaviour is at odds with what social norms expect of parents, and as a result, parents are more likely to experience guilt. In the same vein, compared to educating their children themselves, parents are more reluctant to use AI educational services and less willing to share its benefits with others due to their responsibility attribution. Thus, we propose:

Individuals' attribution of taking children's education as their responsibility mediates the impact of educational approach on individual responses, including (a) guilt, (b) valuation and (c) WOM.

#### The moderating role of locus of causality

Prior research has accumulated abundant evidence on how attribution can influence individuals' emotions and behaviours (Hur et al., 2015; Weiner, 1986). Specifically, locus of causality is one of the attributions often employed by individuals. According to attribution theory, individuals attribute the causes of outcomes to either internal factors (within the person, e.g., lack of ability or effort) or external factors (outside the person, e.g., luck or shortage of time). These attributions influence their emotions, motivations and future behaviours. In the context of tutoring, parents' ability is an essential factor in their choice of educational approach, as some parents may be unable to tutor their children due to a lack of ability or skills.

Individuals' responses to AI are not entirely immutable. Individuals are more likely to adopt AI for tasks that require “ability” (Waytz & Norton, 2014). In terms of tutoring their children, if parents believe that they are not capable of tutoring their children and thereby resort to AI educational services, this can enable their children to obtain better results. In this case, the use of AI educational services provides these less‐competent parents with a justification for effectively substituting themselves. It also helps avoid the negative perception that they are not good parents. Therefore, parents are more willing to spread the benefits of using AI educational services to others when they feel they lack the ability to educate their children. Thus, we predict:

Locus of causality moderates the effect of the educational approach on WOM. Specifically, when individuals lack the ability to tutor their children (intrinsic reason), they are more inclined to spread the WOM about using AI educational services compared to parental engagement. However, in the control condition, individuals are less willing to promote the benefits of using AI educational services for their children compared to parental engagement.

#### The moderating role of conformity

AI educational services are in the burgeoning process of being promoted to the mass individuals, and individuals need a solid reason to use them. Conformity refers to the phenomenon where individuals' behaviour and attitude are influenced by others, causing them to align with the majority of people (Lascu et al., 1995).

Prior research finds that individuals often refer to the information of others before purchasing and using services (Babic‐Rosario et al., 2016; Tucker & Zhang, 2011). Conformity helps individuals in a society become more receptive to a service (Seiler et al., 2014). Behavioural research has shown that conformity provides individuals with social confirmation that a behaviour is justified, leading them to emulate similar behaviour, such as adopting a similar new technology service (Sun, 2013). Conformity information also helps to break social norms and alleviate the negative emotions and behaviours caused by social norms.

In the context of AI educational services, individuals have limited understanding of AI given its nascent nature and the accompanying higher uncertainty about its use. Therefore, knowing that others are also using it can help reduce the perceived risk of adoption. For individuals who choose AI educational services, conformity information will also provide them with social confirmation that their chosen educational approach is not at odds with the social norm of being a responsible parent, thereby relieving them of the concerns about not fulfilling their parental responsibilities. This confirmation is likely to lead to more positive responses towards AI educational services, such as increased WOM about their educational approach to others. In line with this reasoning, we propose that conformity information increases the WOM of individuals who use AI educational services. Thus, we predict:

Conformity moderates the effect of the educational approach on WOM. Specifically, when the conformity cue is absent, individuals are less willing to spread the WOM about using AI educational services compared to parental engagement. However, these differences are diminished when the conformity cue is present.

### Overview of the present research

**FIGURE 1:**
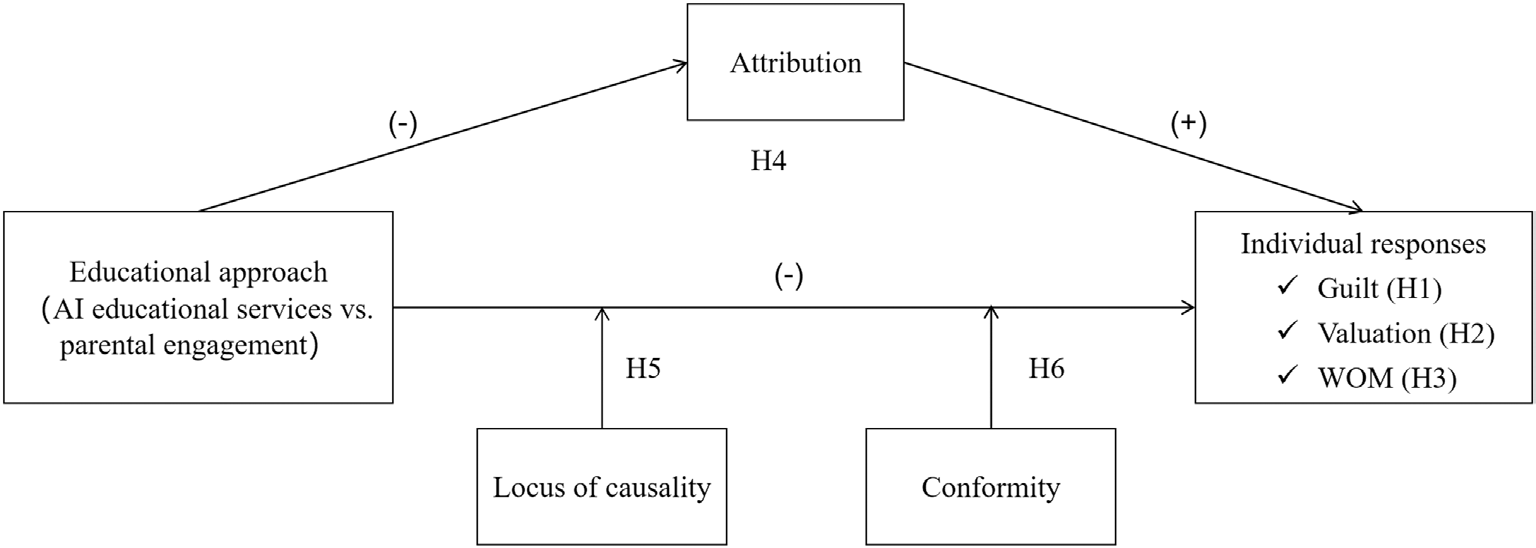
Conceptual framework.

The present research examined six hypotheses through a series of five studies to explore how individuals' educational approaches (parental engagement vs. AI educational services) influence their guilt and downstream behaviours, namely valuation and WOM. We employed experimental designs, a well‐established and widely accepted methodology in psychology, to test our hypotheses. Scenario‐based stimuli are commonly used in experiments to enhance participant engagement and ecological validity (e.g., Celhay & Luffarelli, 2024; Christensen et al., 2024; Liu et al., 2024; Uziel & Seemann, 2025). This approach has been consistently documented in recent studies published in leading psychology journals (e.g., *Journal of Applied Psychology*, *British Journal of Psychology*, *Journal of Consumer Research* and *Journal of Consumer Psychology*), thereby aligning with the field's methodological standards. Furthermore, we utilized randomization in our experimental design to minimize the influence of individual‐difference variables such as gender, identity, age and other demographic characteristics on the effects of our manipulated factors (Kuehl, 2000; Raymond et al., 2023). Studies 1a and 1b established the baseline between educational approach and guilt, as well as valuation, examining H1 and H2. In addition to guilt and valuation, Study 2 also examined the effect of educational approach on WOM, testing H1, H2 and H3. Furthermore, Study 2 investigated whether attribution mediates the relationship between educational approach and guilt, valuation and WOM, examining H4. Study 3 further explored whether the locus of causality (intrinsic attribution vs. control) reverses the effect of educational approach on WOM, testing H5. Study 4 examined the moderating role of conformity in the relationship between educational approach and individuals' downstream WOM behaviour, examining H6. We provide an overview of these five studies in the framework below (see Table 1 and Figure 1).

**TABLE 1:** Overview of studies.

Study	Design	Scenario	Objectives
1a	2 (educational approach: parental engagement vs. AI) between‐subjects design	Tutoring homework	Examining H1 and H2
1b	2 (educational approach: parental engagement vs. AI) between‐subjects design	Tutoring homework	Examining H1 and H2
2	2 (educational approach: parental engagement vs. AI) between‐subjects design	Tutoring writing	Examining H1, H2, H3 and H4
3	2 (educational approach: parental engagement vs. AI) × 2 (locus of causality: intrinsic vs. control) between‐subjects design	Tutoring homework	Examining H5
4	2 (educational approach: parental engagement vs. AI) × 2 (conformity cue: presence vs. absence) between‐subjects design	Tutoring writing	Examining H6

## STUDY 1A

The primary goal of Study 1a is to examine whether individuals are more likely to feel guilty about using AI educational services to educate their children compared to parental engagement (H1). Additionally, Study 1 further explores how the educational approach influences individuals' valuation, examining H2.

### Method

#### Participants and design

This study employed a single factor 2 (educational approach: parental engagement vs. AI) between‐subjects design. Guilt and valuation served as the dependent variables. G*Power^1^ analysis suggested at least 171 participants were required to achieve a power of 0.9 and detect a medium effect size (*f* = 0.25, Faul et al., 2007). A total of 200 participants were recruited via the Credamo online platform. Nine cases with scores exceeding ±2.5 standard deviations were excluded in accordance with Miller (1991) and Rousseeuw and Leroy (1987), resulting in a final sample of 191 participants (139 female, 52 male, *M*_age_ = 32.13, SD = 8.53, age range = 18–59 years old). All participants received monetary compensation.

##### Educational approach

**FIGURE 2:**
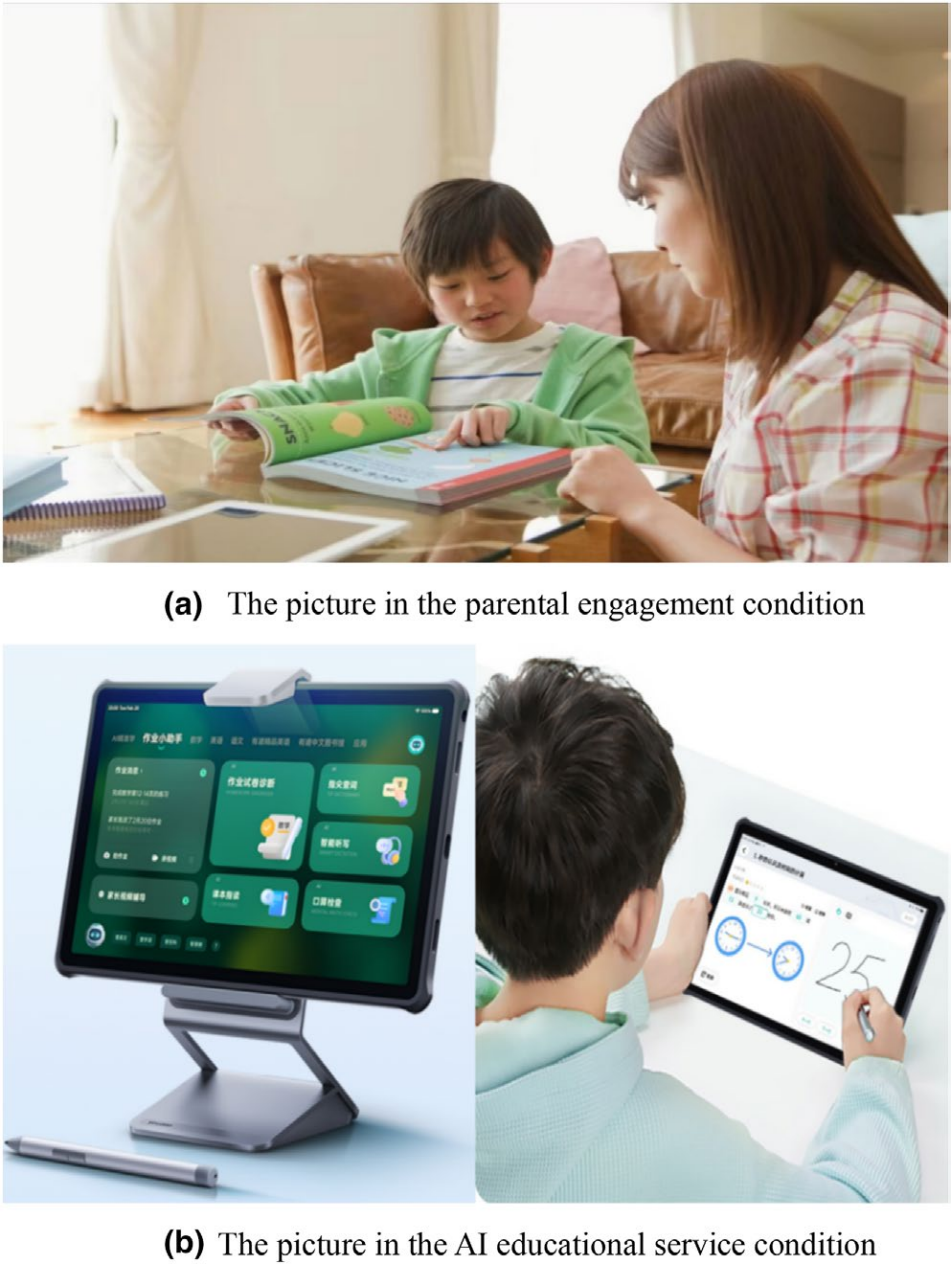
(a) The picture in the parental engagement condition. (b) The picture in the AI educational service condition.

The scenario in this study was tutoring homework. The instruction was: “Imagine that you are a parent and your child needs help with homework every day.” Then, the participants were randomly assigned to two educational approach conditions. The condition of the participant taking on the educational approach was “You help your child with homework every day.” with Figure 2a. The condition of the participant adopting the AI educational service approach was “You buy an AI educational service to help your child with homework every day.” with Figure 2b.

##### Guilt and valuation

An item adapted from Kim and Yoon (2021) was used to measure guilt, providing a straightforward and intuitive test, “If you (omit/use an AI educational service to) help your child with homework every day, do you feel guilty about your child?” (0 = not at all, 10 = very much). An item adapted from O'Donnell and Evers (2019) was used to measure valuation, “How much do you think your tutoring efforts/AI products service are worth in terms of money?_____yuan/month (please fill in the specific amount)”.

### Results

We used an independent sample *t*‐test for guilt and valuation, and the results showed that, compared to educating their children themselves, parents experienced more feelings of guilt and perceived a lower valuation when using AI educational services (Table 2).

**TABLE 2:** The main effect of educational approach on variables in Study 1a.

	Parental engagement	AI educational services	*t*	*p*
Guilt	2.09 (1.58)	2.74 (1.81)	2.67	.008
Valuation^a^	2097.99 (2217.96)	1219.31 (1154.32)	3.41	.001

^a^ The variable exhibited pronounced right skew (overall skewness = 3.28, SE = 0.18). Consequently, we applied a base‐10 log transformation after adding a constant of 1 to accommodate zero values (i.e., log_10_[Valuation +1]). Reanalysis using the transformed variable confirmed that the effect of the educational approach remained statistically significant and in the same direction, *t*(189) = 3.24, *p* = .003.

### Discussion

Study 1a verified H1 and H2. First, it demonstrated that parents who used AI educational services to tutor their children experienced more guilt about their children than those tutored by themselves. In addition, the educational approach further influenced individuals' downstream behaviour, namely, valuation. Parents perceived more value in educating their children themselves than in using AI educational services.

We used both pictures and words to manipulate the educational approach in Study 1a. To exclude the influence of picture content and examine the robustness of our findings, we will only employ words to manipulate the educational approach in Study 1b. In addition, we will control parental status in Study 1b.

## STUDY 1B

Study 1b has two primary objectives. First, we change the manipulation of the educational approach to examine H1 and H2 again. Second, we aim to exclude the effect of parental status on guilt and valuation.

### Method

#### Participants and design

This study employed a single‐factor 2 (educational approach: parental engagement vs. AI) between‐subjects design. Guilt and valuation served as the dependent variables. A total of 200 participants (135 female, 65 male, 156 participants had children, *M*_age_ = 31.44, SD = 8.45, age range = 18–69 years old) were recruited via the Credamo online platform, with monetary payment as compensation. All participants correctly answered the attention check question, so no participant was excluded.

##### Educational approach

The scenario in this study was tutoring homework, the same as Study 1a. The only difference was we didn't provide pictures in the two conditions.

##### Guilt and valuation

Three items adapted from Kim and Yoon (2021) were used to measure guilt, “Do you feel guilty/bad/remorse about your children?” (0 = not at all, 10 = very much), Cronbach's *α* = .96. We used the same measure for valuation as in Study 1a. In addition, the presence or absence of children (yes/no) was measured as a control variable. Lastly, we included an attention check in the questionnaire to ensure the quality of the data collected. “The scenario in the questionnaire is: ____. A. Listen to music B. tutoring homework C. Listen to the news D. Tell jokes.”

### Results

Although the target individuals of AI educational services are parents with children, we believe that adult individuals, regardless of whether they currently have children, could also be potential users of AI educational services. Therefore, we wanted to understand the psychology and behaviour of all individuals, regardless of whether or not they have children.^2^ We conducted a one‐factor (educational approach: parental engagement vs. AI) ANOVA on guilt and valuation. The results showed that the main effect of educational approach on guilt was significant, *F*(1, 198) = 14.07, *p* < .001, $\eta_{p}^{2}=.07$; the main effect of educational approach on valuation was also significant, *F*(1, 198) = 18.24, *p* = .001, $\eta_{p}^{2}=.08$. Compared to engaging in educating their children themselves (*M*_guilt_ = 3.79, SD = 2.47; *M*_valuation_ = 2494.11, SD = 2530.90), individuals experienced more feelings of guilt and perceived less value when using AI educational services (*M*_guilt_ = 5.23, SD = 2.94; *M*_valuation_ = 1219.62, SD = 1581.27).

### Discussion

Study 1b verified H1 and H2 again; the results of Study 1b held true as those of Study 1a, where only words were used in the educational approach manipulation. Parental individuals who used AI educational services to tutor their children felt more guilty about their children than those engaged in tutoring themselves. Additionally, parents perceived more value in engaging in educating their children themselves than in using AI educational services. Furthermore, Study 1b excluded the influence of parental status on guilt and valuation.

## STUDY 2

Study 2 has three primary goals. First, in addition to guilt and valuation, we examine the impact of educational approach on WOM. Second, we explore the mediating role of attribution between educational approach and guilt, as well as downstream behaviours. Third, we aim to rule out an alternative explanation that parents' feelings of guilt and negative responses to AI educational services are due to their concern about the inability of AI educational services to tutor their children.

### Method

#### Participants and design

This study employed a single factor (educational approach: parental engagement vs. AI) between‐subjects design. Guilt, valuation and WOM served as dependent variables. A total of 200 participants (122 female, 78 male, 157 participants had children, *M*_age_ = 32.53, SD = 9.84, age range = 18–69 years old) were recruited via the Credamo online platform with monetary payment as compensation.

##### Educational approach

The scenario was tutoring writing. The instruction was: “Imagine that you are a parent and your child needs help with writing.” The participants were randomly assigned to one of two conditions and asked to indicate their feelings and behaviours. In the condition where participants took on the responsibility of tutoring their children themselves as an educational approach, the instruction was: “You help your children with their writing every day.” In the condition where participants used AI educational services as the educational approach, the instruction was: “You buy a ‘Writing Expert’ AI educational service to help your children with their writing every day.”

##### Attribution, guilt and WOM

To measure attribution, three items were adapted from Leo and Huh (2020), “To what extent do you think you/educational services are responsible for tutoring your child's writing?” on a scale from 0 (no responsibility at all) to 10 (fully responsible), “You believe that educating children is the responsibility of you/AI educational services.”, and “You/educational services need to take responsibility for educating children.” on a scale from 0 (strongly disagree) to 10 (strongly agree), Cronbach's *α* = .86. The items for measuring guilt (Cronbach's *α* = .93) and valuation were the same as in Study 1b. Three items adapted from Zeithaml et al. (1996) were used to measure WOM, including “You want to talk to people about the way you/AI educational services tutor children's education,” “You are willing to recommend to your family and friends the way you/AI educational services tutor children's education,” and “You are willing to recommend the way you/AI educational services tutor children's education to people who ask for your advice.” Participants rated these items on a scale from 0 (strongly disagree) to 10 (strongly agree), Cronbach's *α* = .86. In addition, the presence or absence of children (yes/no) and experience with similar AI educational services (0 = completely no; 10 = frequently use) were used as control variables. The perception of AI educational services' ability was also measured to rule out possible explanation, “Do you think current AI educational services can help you tutor your child’ writing?” (0 = completely no; 10 = completely yes).

### Results

First, the results of an independent sample *t*‐test showed no significant difference between the two groups in terms of familiarity with AI educational services (*M*_parental engagement_ = 6.60, SD = 2.74 vs. *M*_AI_ = 6.90, SD = 2.34), *t*(198) = 0.83, *p* = .406. Next, we conducted a one‐factor (educational approach: parental engagement vs. AI) ANCOVA on guilt, valuation, WOM, attribution and perception of AI educational services capability. Our analysis showed consistent results regardless of whether we used the data of parent participants or all participants, similar to the findings in Study 1b. Specifically, compared to educating their children by themselves (parental engagement), parents adopting AI educational services experienced more feelings of guilt towards their children, perceived less value and were less willing to recommend their educational approach to others. Meanwhile, those in the parental engagement condition were more likely to view tutoring children as their responsibility than those in the AI educational services tutoring condition (Table 3).

**TABLE 3:** The main effect of educational approach on variables in Study 2.

	Parental engagement	AI educational services	*F*	*p*	*η*_p_^2^
Guilt	2.32 (2.31)	3.09 (2.35)	4.18	.043	.03
	2.14 (2.21)	3.66 (2.79)	17.82	<.001	.08
Valuation	968.14 (780.09)	477.83 (547.20)	21.14	<.001	.12
	1055.19 (1036.75)	448.75 (508.54)	27.11	<.001	.12
WOM	8.23 (1.41)	7.83 (1.65)	4.77	.030	.03
	8.11 (1.45)	7.79 (1.67)	3.11	.079	.02
Attribution	8.79 (0.86)	6.27 (2.50)	87.92	<.001	.36
	8.77 (0.90)	6.11 (2.54)	110.61	<.001	.36
Perception of AI's capability	7.42 (2.63)	7.53 (2.28)	0.20	.655	/
	7.05 (2.73)	7.42 (2.35)	0.47	.496	/

*Note*: The first line for each variable showed the analysis result of participants with children, and the second line showed the result of all participants.

In addition, individual perception of AI educational services capability showed that, even after controlling for the experience with similar AI educational services, individuals believed that current AI educational services could help them with their children's writing, regardless of whether they were in the parental engagement condition or the AI educational services condition. This result allowed us to exclude the alternative explanation that differences in individuals' guilt, valuation and WOM were due to their perception of AI educational services' capabilities.

To examine the mediating effect of attribution on the relationship between educational approach and guilt, valuation and WOM, we used Hayes (2022) bootstrapping procedures (Model 4, 5000 bootstrap samples, 95% bias‐corrected confidence intervals [CI]) with the educational approach as the independent variable (X), attribution as the mediator (M), and guilt, valuation and WOM as the dependent variables (Y). Our results revealed that, as predicted, attribution mediated the relationship between educational approach and the dependent variables, including guilt, valuation and WOM, which, to a certain extent, explained why individuals were more likely to experience feelings of guilt, perceived less value and be less likely to spread the benefits when using AI educational services to tutor their children, compared to engage in tutoring by themselves (Table 4).

**TABLE 4:** The mediating effect of attribution between educational approach and variables in Study 2.

DV	Effect	SE	95% CI
Guilt	0.6116	0.3113	0.0244, 1.2555
Valuation	−183.9280	57.3749	−303.1121, −79.2954
WOM	−0.8910	0.2299	−1.4199, −0.4955

### Discussion

Study 2 provided further evidence for our findings. When individuals use AI educational services to tutor their children, they experience significantly more feelings of guilt, perceive less value and are less willing to spread the benefits, compared to when they engage in educating their children themselves. These findings support H1, H2 and H3. In addition, we excluded the presence of children as a variable, and the results remain consistent regardless of whether participants were parents or not. Therefore, we did not include this variable in the analysis of subsequent studies.

Study 2 also explored the mediating role of attribution between educational approach and individuals' feelings of guilt and downstream behaviours. When parents use AI educational services for their children's education, they perceive that they are not taking their parental responsibility for their children's education. Thus, parents experience more feelings of guilt, perceive less value in using AI educational services and are less likely to spread the benefits to others, compared to engaging in education by themselves, supporting H4. We also ruled out a potential explanation that parents feel more guilty and exhibit negative behaviours when using AI educational services to tutor their children compared to parental engagement due to thinking the AI educational services are less capable.

## STUDY 3

Study 3 manipulates the locus of causality to verify its moderating role. Whether a parent can provide educational assistance to their children depends on their own ability. Taking homework tutoring as an example, it requires parents to have a certain level of knowledge and tutoring skills. When parents lack these abilities, this intrinsic factor may increase their willingness to adopt AI educational service and make them more likely to recommend this service to others. In Study 3, we explore whether this intrinsic reason causes differences in WOM, verifying H5.

### Method

#### Participants and design

Study 3 employed a two‐factor 2 (educational approach: AI vs. parental engagement) × 2 (locus of causality: intrinsic vs. control) between‐subjects design. WOM served as the dependent variable. A total of 390 participants (245 female, 145 male, *M*_age_ = 31.09, SD = 8.90, age range = 18–75 years old) were recruited via the Credamo online platform with monetary payment as compensation.

##### Locus of causality

The scenario involved tutoring homework as follows: “Imagine that you are a parent, and your child needs help with homework every day”. Participants were randomly assigned to one of two conditions, the intrinsic group or the control group. The instruction in the intrinsic group was: “Although you have time, you are short of necessary ability in tutoring.” The instruction in the control group did not include any additional information. The rest of the procedure and the WOM measure were the same as in Study 2.

### Results

**FIGURE 3:**
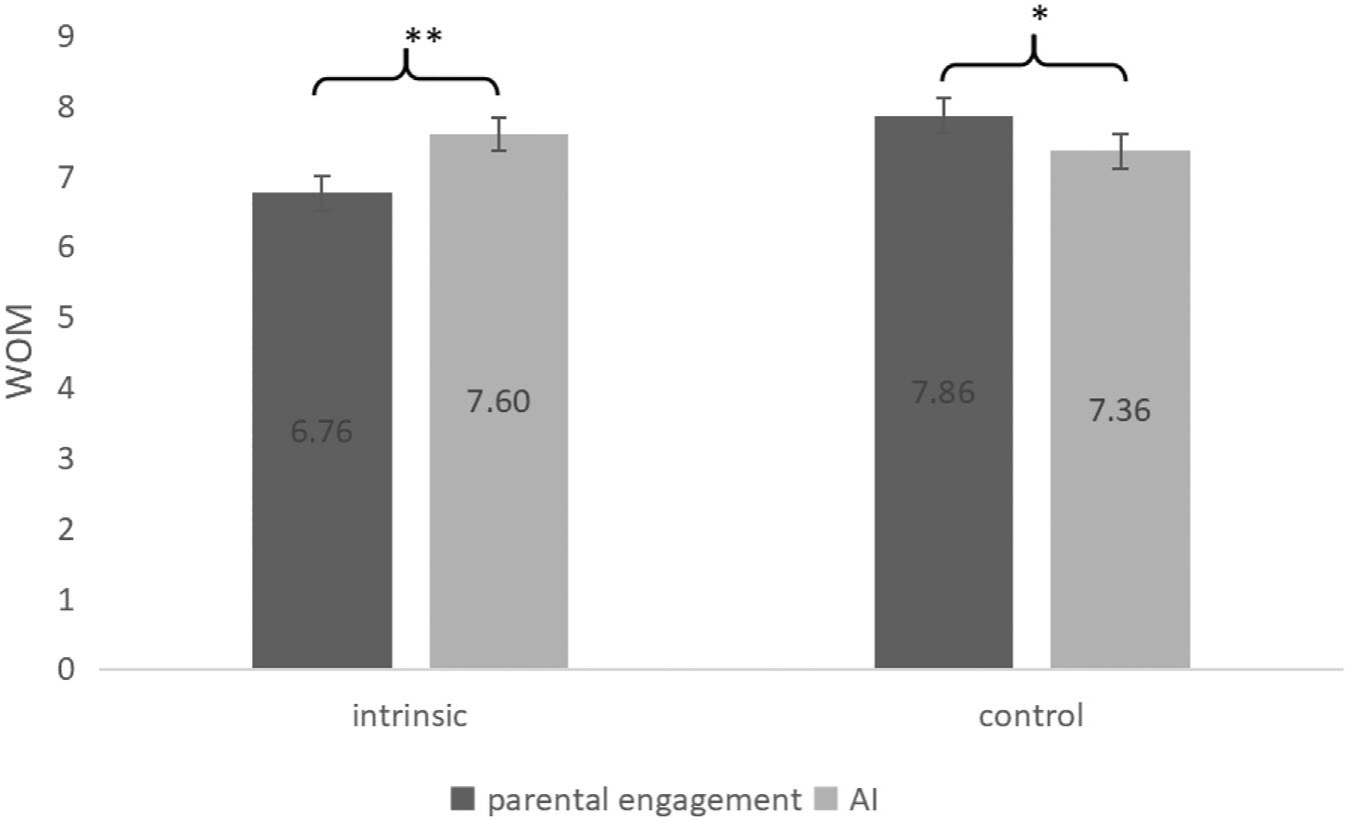
The interaction of educational approach and locus of causality on WOM in Study 3.

Study 3 used a two‐factor 2 (educational approach: AI vs. parental engagement) × 2 (locus of causality: intrinsic vs. control) ANCOVA on WOM. The results showed that, even after controlling for experience with similar AI educational services (*p* < .001), the main effect of locus of causality on WOM was significant, *F*(1, 385) = 6.16, *p* = .014, $\eta_{p}^{2}=.02$. Compared with the control condition (*M* = 7.61, SD = 1.62), parents in the intrinsic condition spread significantly less positive WOM to others (*M* = 7.19, SD = 1.96). The interaction effect of educational approach and locus of causality on WOM was significant, *F*(1, 385) = 11.65, *p* = .001, $\eta_{p}^{2}=.03$. Specifically, when parents lacked the ability to help with children's homework (and used AI educational services for intrinsic reasons), compared with engaging in tutoring children themselves (*M* = 6.76, SD = 2.29), parents who used AI educational services spread significantly more positive WOM to others (*M* = 7.60, SD = 1.47), *p* = .006. However, in the control condition, compared with engaging in tutoring children themselves (*M* = 7.86, SD = 1.23), parents who used AI educational services spread significantly less positive WOM to others (*M* = 7.36, SD = 1.90), *p* = .04 (Figure 3).

### Discussion

Parents generally consider educating children to be their responsibility, but when they are unable to tutor their children for a certain intrinsic reason, AI educational services can be employed to assist them. Study 3 examined whether using AI educational services due to intrinsic reasons could improve their WOM. The results showed that when parents are unable to help with homework due to intrinsic reasons (lack of ability), they spread significantly more positive WOM to others when using AI educational services than by engaging in tutoring themselves, supporting H5.

## STUDY 4

Study 3 examined how to improve individuals' WOM regarding the use of AI educational services for intrinsic reasons. In Study 4, we shift our focus to explore whether the actions of others (conformity) can enhance individuals' WOM regarding the use of AI educational services.

### Method

#### Participants and design

Study 4 employed a 2 (educational approach: AI vs. parental engagement) × 2 (conformity cue: presence vs. absence) between‐subjects design, with WOM as the dependent variable. A total of 400 participants (250 female, 150 male, *M*_age_ = 32.93, SD = 10.15, age range = 18–69 years old) were recruited via the Credamo online platform with monetary payment as compensation.

##### Conformity cue

All participants were randomly assigned to one of two groups: a conformity cue presence group and a conformity cue absence group. Participants in the conformity cue presence group were told, “your parental friends are helping their children's writing by themselves/using AI educational services,” while those in the conformity cue absence group were not shown any information. The educational approach, WOM and control variable were the same as in Study 2.

### Results

**FIGURE 4:**
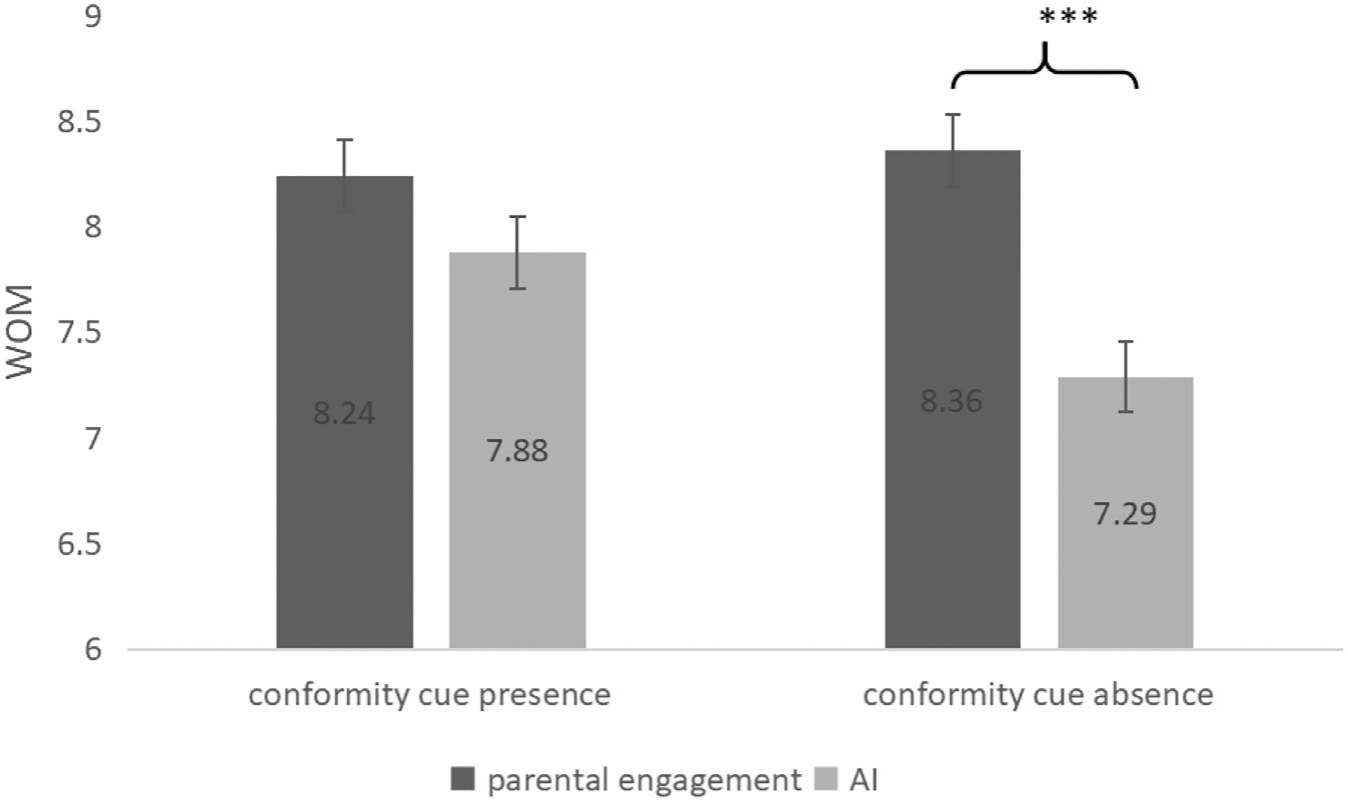
The interaction of educational approach and conformity cue on WOM in Study 4.

None of the control variables influences WOM; hence, no further analysis was performed. We conducted a 2 (educational approach: AI vs. parental engagement) × 2 (conformity cue: presence vs. absence) ANOVA on WOM. The results showed that the main effect of educational approach on WOM was significant, *F*(1, 396) = 17.23, *p* < .001, $\eta_{p}^{2}=.04$. Compared with tutoring their children themselves (*M* = 8.30, SD = 1.10), parents who used AI educational services spread significantly less WOM to others (*M* = 7.59, SD = 2.19). The interaction effect of educational approach and conformity cue on WOM was also significant, *F*(1, 396) = 4.09, *p* = .044, $\eta_{p}^{2}=.01$. In the conformity cue presence condition, the difference in WOM between engaging in educating children themselves (*M* = 8.24, SD = 1.23) and using AI educational services (*M* = 7.88, SD = 1.94) was not significant, *p* = .133. In contrast, in the conformity cue absence condition, compared to engaging in educating children themselves (*M* = 8.36, SD = 0.94), parents who used AI educational services (*M* = 7.29, SD = 2.39) spread significantly less WOM to others, *p* < .001 (Figure 4).

### Discussion

The results of Study 4 showed that conformity plays a moderating role in the influence of educational approach on WOM. This study revealed that providing conformity information can significantly increase WOM behaviour among individuals who use AI educational services.

## GENERAL DISCUSSION

Through a series of five studies and varied educational contexts, this research is the first to document how parent individuals' educational approach impacts their guilt and downstream behaviours. Research shows that although AI services have made extraordinary advancements in terms of functionality (Labadze et al., 2023), individuals still show resistance to using them. In this research, we found that the educational approach of using AI educational services leads to individuals' increased feelings of guilt, perceived lower valuation and reduced WOM compared to a parental engagement approach. This intriguing finding holds significant implications for the promotion of AI educational services and for leveraging AI technology for AI and human interaction at large.

We further shed light on why such effects exist through an attribution‐based account as the underlying process. Specifically, influenced by a widely accepted social norm, individuals generally believe that educating children is an expression of parental responsibility and love, leading to their relatively negative responses to using AI educational services. This result is similar to that of Garcia‐Rada et al. (2022), where individuals expect to express their love by putting more effort into kids' caregiving.

Meanwhile, we elucidated that the locus of causality and conformity moderate the effect of the educational approach on downstream WOM behaviour. WOM serves as a dual purpose for individuals engaging in these exchanges: it not only helps them express their self‐value but also reinforces their identity within the community (Shao et al., 2025). Thus, when parents lack the ability (an intrinsic reason) to tutor their children, they are more inclined to spread WOM about using AI educational services compared to engaging in education themselves. Moreover, conformity helps individuals engage in social confirmation through external information about others. It provides justification for using AI educational services and thus increases individuals' WOM for AI educational services.

## THEORETICAL IMPLICATIONS

This research makes three main contributions. First, to the best of our knowledge, our research is the first to explore individuals' emotional dark side and negative downstream behaviours arising from the use of AI educational services. Our research challenges the common assumption that improving the performance of AI services will make individuals more willing to use them (e.g., Castelo et al., 2019; Kim et al., 2022; Longoni et al., 2019). It reveals that the emergence of negative emotions and subsequent behaviours is not due to the subpar performance of AI educational services, but rather to entrenched social norms among individuals. Given that technology providers should not ignore individual experience while developing new services (Becker & Jaakkola, 2020; Moore, 2014), our research highlights the importance of considering individual psychology in technological innovation development. More specifically, it unravels a seemingly paradoxical situation of why parents, despite recognizing the benefits of AI educational services for their children, remain hesitant to use them. Our research demonstrates that this reluctance stems from the complex interplay between technological efficacy and social norms.

Second, we explore individual preferences between AI educational services and parental engagement and elucidate the attribution behind these choices. The results of Study 2 suggest that individuals are more inclined towards parental engagement than using AI educational services, given their ingrained social norms of parental responsibility attribution– parents believe they should take responsibility to engage in educating their children themselves rather than using AI educational services. This attribution mechanism reveals an intriguing yet hitherto under‐examined psychological process that impacts individuals' responses towards AI educational services. Hence our research offers critical psychological insights into AI educational services usage, expanding existing theories of individual behaviour and attribution theory, thus providing a new perspective on the reluctance to adopt AI educational services.

Third, our research delves into a spectrum of diverse individual responses to AI educational services, encompassing feelings of guilt, valuation and WOM. This comprehensive approach provides a holistic view of individual responses, which is critical to understanding the multifaceted nature of individual involvement in AI education. In addition, our research explores the potential of WOM in promoting AI educational services, highlighting psychological factors such as intrinsic attribution and conformity. These factors provide unique insights into fostering positive WOM and acceptance of AI educational services. In doing so, our research enriches academic dialogue at the intersection of AI, education and individual psychology, providing a more panoramic and enriched understanding of the use of AI educational services.

## PRACTICAL IMPLICATIONS

This research provides important practical implications for practitioners in the AI educational service industry. First, managers in the AI educational service sector need to understand the adverse parental responses to AI educational services, which are rooted in social norms surrounding parental responsibility rather than the services themselves. By emphasizing parental love and responsibility, practitioners can reduce individuals' negative perceptions of using AI educational services and increase their acceptance. Understanding individual psychology can help managers better position these services in the market, promote positive WOM among individuals and foster synergies between parents and AI services to improve off‐campus learning outcomes.

Second, managers can strategically highlight how AI educational services complement parental responsibilities, thereby enhancing perceived value and stimulating positive WOM. For instance, Khan Academy illustrates how their AI‐powered learning guide, Khanmigo, provides personalized tutoring across multiple subjects like physics, computer science and mathematics and writing, offering interactive experiences and real‐time feedback to student users. These powerful illustrations could not only mitigate individuals' negative feelings of guilt, meet the emotional needs and social expectations of individuals, but also foster broader adoption of AI educational services and encourage proactive WOM and advocacy among peers, thereby enhancing the adoption of AI in educational practices.

Third, managers could provide parents with conformity information that reflects the adoption of AI educational services within their social circles. Such information acts as a social signal, alleviating parental anxieties regarding their parental responsibilities. By showing that other parents are utilizing AI educational services for their children, it breaks traditional norms of parental responsibility and encourages broader adoption. Consequently, it enhances individuals' WOM and drives future adoption of AI educational services, with significant implications for the growth of the burgeoning AI‐in‐Education industry and the potential higher adoption of AI to enhance human well‐being as a whole.

## LIMITATIONS AND DIRECTIONS FOR FUTURE RESEARCH

This research still has some limitations and potential for future enhancement. Firstly, we acknowledge that the findings of this research are specific to the context of Chinese and Asian culture. Confucian norms prevalent in East Asian contexts may cause the delegation of educational responsibilities to AI to be more readily interpreted as parental irresponsibility compared with Western cultural norms. Lin et al. (2023) indicate that guilt is more pronounced among parents in collectivistic cultures. In Western cultural contexts, parents typically emphasize the cultivation of autonomy and therefore exhibit comparatively lower levels of direct involvement than their East Asian counterparts. Nonetheless, highly education‐oriented parents—particularly those from the middle class—may still engage to a moderate extent. We therefore expect the observed effect to be attenuated rather than eliminated in Western samples. Therefore, exploring variations in individuals' responses across cultures presents an interesting avenue for future research.

Secondly, parental involvement and technology usage vary significantly depending on the child's age, learning goals and context. This research primarily focuses on AI educational services related to tutoring in writing and homework. Although these tasks are representative of many current parent–child interactions with AI tutors, they do not capture emerging applications such as exploratory learning, language skills, test preparation and interest‐driven topics. Consequently, the observed effects may not generalize to contexts in which AI is deployed primarily for open‐ended discovery or skill acceleration rather than task completion. Future research should extend the stimulus set to encompass these domains and thereby test the robustness of our findings.

Thirdly, we acknowledge that relying solely on self‐reported Likert ratings of guilt and WOM may be susceptible to social‐desirability bias, particularly in the highly valenced context of “being a good parent”. In addition, we acknowledge that our research may not fully capture the complexity of these interactions, especially regarding the specific topics parents choose to share or their motivations for doing so. Therefore, future research should incorporate behavioural proxies (e.g., monetary recompense offers to the child, time spent on reparative tasks) or indirect emotion‐elicitation methods (e.g., subtle facial EMG during parent–child interaction, affective priming tasks) to mitigate self‐report biases. Ecologically valid measures (e.g., message framing tasks, behavioural intention tracking, or peer‐sharing simulations) should also be adopted to capture actual WOM behaviour. Finally, longitudinal designs or real‐world deployments of AI services are needed to obtain ecologically valid insights into actual usage patterns.

## AUTHOR CONTRIBUTIONS

**Aiping Shao:** Conceptualization; investigation; methodology; data curation; formal analysis; writing – original draft. **Zhi Lu:** Investigation; funding acquisition; writing – original draft; writing – review and editing; methodology; formal analysis; supervision; project administration; resources. **Stephanie Q. Liu:** Investigation; writing – review and editing; resources. **Yin Shi:** Investigation; project administration. **Wei Lu:** Investigation; funding acquisition; project administration; supervision.

## CONFLICT OF INTEREST STATEMENT

All of the authors have no competing interests.

## ETHICS STATEMENT

This research was approved by the Human Research Ethics Board of Shanghai Jiao Tong University (No. H20230301I).

## Data Availability

The data that support the findings of this study is openly available in the Open Science Framework at https://osf.io/rtcg3/?view_only=d1d5f4097c4f4aa3a7fc698344521127.

## Footnotes

^1^
Similar G*Power analyses were executed for the following studies. We omit them for brevity.

^2^
Additional analyses were conducted to show that there were no significant differences in gender (*M*_parental engagement_ = 1.74, SD = 0.44 vs. *M*_AI_ = 1.61, SD = 0.49) and age (*M*_parental engagement_ = 31.60, SD = 9.15 vs. *M*_AI_ = 31.27, SD = 7.72) between the two groups, *p* > .05. Therefore, gender and age were no longer included in the subsequent analysis. In addition, to exclude the effect of parental status on guilt and valuation, we also analysed the results of parent participants. The results showed that the main effect of educational approach on guilt was significant, *F*(1, 154) = 14.64, *p* < .001, $\eta_{p}^{2}=.09$; the main effect of educational approach on valuation was significant too, *F*(1, 154) = 12.43, *p* = .001, $\eta_{p}^{2}=.08$. Compared to engage in educating their children themselves (*M*_guilt_ = 3.47, SD = 2.39; *M*_valuation_ = 2409.16, SD = 1917.55), individuals experienced more feelings of guilt and perceived less value when using AI educational services (*M*_guilt_ = 5.15, SD = 3.05; *M*_valuation_ = 1388.00, SD = 1695.94). The results were consistent regardless of whether we used data only from parent participants or all participants.
